# “One pastor advised him to stop taking HIV medication”: Promoters and barriers to HIV care among gay, bisexual, and men who have sex with men living with HIV in Ghana

**DOI:** 10.21203/rs.3.rs-4087718/v1

**Published:** 2024-03-22

**Authors:** Gamji Rabiu Abu-Ba’are, Gloria Aidoo-Frimpong, Prince Amu-Adu, Edem Yaw Zigah, Melissa Stockton, Samuel Amuah, Richard Panix Amoh-Otoo, Laura Nyblade, Kwasi Torpey, LaRon E. Nelson

**Affiliations:** Behavioral, Sexual and Global Health Lab, School of Nursing, University of Rochester Medical Center, University of Rochester; Yale AIDS Prevention Program (Y-APT), Yale University; PORSH; Behavioral, Sexual and Global Health Lab, School of Nursing, University of Rochester Medical Center, University of Rochester; University of North Carolina at Chapel Hill; Youth Alliance for Health and Human Rights, YAHR; Youth Alliance for Health and Human Rights, YAHR; RTI International; University of Ghana; Yale School of Nursing

**Keywords:** GBMSM, HIV care, PLHIV, Ghana, HIV stigma, alternative medicine

## Abstract

**Introduction:**

Few studies examine Ghanaian gay, bisexual, and other men who have sex with men (GBMSM) experience with HIV diagnoses and linkage to care. This article provides qualitative accounts of promoters and barriers to care among GBMSM living with HIV in Ghana.

**Methods:**

We recruited and interviewed 10 GBMSM living with HIV in two Ghanaian cities. We transcribed the interviews, coded the data, and used thematic content analysis.

**Results:**

We found that community and healthcare facility (HCF) level HIV and sexual stigma, confidentiality issues, alternative medicine, and substance use remain the key barriers to care. Other barriers include healthcare system issues such as long wait times and economic problems (e.g., health insurance and financial difficulties). Nonetheless, HCF-level factors such as positive experiences with providers, HIV counseling, and detailed medication information facilitate adherence to care among GBMSM.

**Conclusion:**

This study highlights the need for interventions that address linkage to care issues, especially substance use, disinformation, and misinformation among GBMSM and other Ghanaian communities.

## Introduction

Sub-Saharan Africa (SSA) remains the region most severely affected by the HIV epidemic, with around 60 – 70% of the 38.4 million people living with HIV worldwide residing in the region [1]–[3]. In Ghana, an estimated 346,120 people live with HIV, with an overall prevalence rate of 1.7%[4]. Ghana, through its health service agencies and with international support from organizations such as the U.S. President’s Emergency Plan for AIDS Relief (PEPFAR), has made significant strides in controlling the epidemic[5], [6]. As of 2020, an estimated 71% of people living with HIV in Ghana knew their status, 99% received antiretroviral therapy (ART), and 79% attained viral suppression[4]. Ghana has also progressed in HIV testing and counseling, increasing from about 1 million tests in 2016 to 1.8 million in 2020[4]. Additionally, the number of ART sites across the country continuously increased, with the number of people receiving ART also growing dramatically, from 45,000 in 2010 to 250,000 in 2020[7], [8].

Despite the advancement in HIV care, Ghanaian gay, bisexual, and other men who have sex with men (GBMSM) encounter unique barriers that intensify their susceptibility to contracting HIV and lead to inferior HIV outcomes[9]–[12]. Such barriers include insu cient knowledge of HIV, low condom use, low testing, and stigma associated with sexual behavior, HIV, and gender expression [9]–[13]. These barriers have translated into a disproportionately high HIV prevalence among Ghanaian GBMSM in particular (18%), compared to the general population (1.7%)[4], [14]. The HIV prevalence among GBMSM is even higher in metropolitan regions such as Greater Accra (42.2%) and Ashanti (25.4%)[4], [11].

Globally, compared to heterosexual men, GBMSM, especially Black GBMSM, face more significant difficulties in accessing healthcare services, including HIV testing and ART, due to systemic barriers such as poverty, racism, and lack of access to healthcare facilities[15]–[17]. In sub-Saharan Africa, stigma and discrimination, criminalization of same-sex behavior, and lack of culturally competent healthcare providers all hamper access to HIV testing and treatment services[18]–[21]. However, there is a dearth of research on the Ghanaian GBMSM experience with HIV care. The few studies that included GBMSM living with HIV or measured testing and HIV care experiences in Ghana documented high levels of stigma, social isolation, and discrimination due to HIV, sexual behavior, and gender expression as barriers to care[22]–[24]. The criminalization of same-sex sexual practices in Ghana exacerbates an already hostile and unaccepting environment for GBMSM living with HIV, further hindering HIV care [5]–[7], [21], [25]

### The current study:

This study aims to elucidate the GBMSM experience across the HIV care continuum, including testing, receiving a positive diagnosis, initiating treatment, and engaging and adhering to ART. It highlights factors that may act as barriers or promoters to care after diagnosis. These findings are essential to devising interventions to optimize HIV testing and linkage to care among GBMSM.

## Methods

The data analyzed for this manuscript is from the preliminary stage of a randomized controlled trial (RCT) on the efficacy of a multi-level intervention to decrease intersectional HIV, gender nonconformity, and sexual stigma and to increase HIV testing among GBMSM in Ghana[9], [20], [26], [27]. We present a brief overview of the methods; a more comprehensive description has been published elsewhere [9], [20], [26], [27].

We employed in-depth interviews (IDIs) to gain insights into GBMSM experience across the HIV care continuum, including testing, receiving a positive diagnosis, initiating treatment, engaging, and adhering to ART. Participants responded to questions about the circumstances around their HIV diagnoses (experience at a diagnostic facility, their reaction to a positive test, and how they accepted their status). Participants also talked about their experience with care (e.g., medication access and adherence) and the facilitators and barriers to HIV care at all socio-ecological levels (the interpersonal, intra-personal, community, and healthcare facility levels).

### Sampling and Data Collection

Two community partner organizations utilized convenience and snowball sampling methods in Accra and Kumasi to recruit 10 adult GBMSM living with HIV in Ghana. The participants self-disclosed they were living with HIV, 18 years or older, male at birth, identified as men, and had engaged in sexual activity with another man within the previous six months. The interviewers received training in data collection and human subject research and identified themselves as gay or bisexual men from the respective cities. The interviewers conducted the IDIs at their respective organizations. All interviews were conducted in English, audio recorded, and transcribed. The research team reviewed the audio files of the initial interviews to ensure consistency, provided feedback to the interviewers, and revised the guides when necessary.

#### Data analysis.

The data were coded line by line by seven members of the research team and partner organizations. They began by reviewing the transcripts to identify relevant concepts on HIV care, which they then used to create a codebook. Each transcript was independently coded by two coders using the codebook and resolved any discrepancies by discussing and reaching a consensus in a meeting. They grouped the resulting codes into broader categories representing the participants’ experiences and perspectives on diagnoses and care.

## Results

We identified and analyzed the relationship and commonalities between the participants’ responses related to HIV diagnoses and experiences with care engagement. The themes that emerged included (a) experiences with HIV diagnosis, (b) promoters for care engagement, and (c) barriers to care engagement.

### 1. Experiences with HIV diagnoses

All participants in the study had an emotional reaction to testing and receiving a positive HIV result. They expressed initial shock and distress, denial, or nonacceptance of test results.

#### Shock and distress.

Participants reported experiencing distress before testing. They also experienced shock and distress after receiving a positive HIV test result. Experiences before testing include fear of positive test results and anticipation of negative consequences.

“Being HIV positive is not something you should be happy with, so when I was going for the test, I was having these panic attacks and anxiety.” Participant

“I could remember when I went to do my HIV test; it was very scary; I was like, wow, my gosh! What if I am HIV positive? What will happen next?” So, when I did my test, the results didn’t even come, but I was crying. I was crying, and I was so scared because I was like. Eish! So, if I am HIV positive, would I die? I would die because I can’t take medicine for the rest of my life. A whole lot was on my mind. Even within those five to ten minutes, I was sweating because I didn’t know what the results would be, so I was like, wow, Oh God help me, don’t let it be that I am HIV positive.” Participant

Participants were afraid of being positive, in part because they did not know what the future would bring; they were afraid of dying from the disease and afraid of having to take medication for the rest of their lives. After testing, many participants felt broken and distressed about testing positive for HIV:

“It was very appalling, very bad to find out that I am HIV positive. It kills the inner system in you.” Participant

“I was totally broken down. I cried, how did it happen, how, it’s a moment that… I can’t forget that moment.” Participant

#### Denial of test results.

GBMSM who tested positive initially struggled to accept their HIV status, with several doubting or disbelieving the HIV test result.

“I went to [Facility NAME] first and was diagnosed. I didn’t believe it; then, I went back to [Facility NAME] to redo the test, which was the same result. So, because of that, I realized that it’s true, and I really have it.” Participant

However, GBMSM were able to accept their status with counseling gradually:

“No, I wasn’t expecting it, and it was very difficult for me to accept, but through counseling, I was able to be myself.” Participant

“Because I didn’t accept my status for two weeks, and she (nurse) was still counseling me, and she kept calling me to come to the facility. Just to have my confirmation test.” Participant

#### Feeling/fear of judgment or stigmatization.

After diagnosis, some participants felt judged by providers for testing positive or not knowing much about HIV. Others begin to develop issues around trust, especially with disclosing status to people such as family members:

“The discrimination started from the hospital because it got to a time when the lady (nurse) spoke to me in a way that I feel bad. I didn’t know anything about HIV, and even how to use a condom was a problem for me because my education was not enough. I didn’t get people to educate me more or know certain things about preventing yourself from getting the infection, so the discrimination started from there. The lady started to speak to me anyhow (condescending or disrespectful).” Participant

“Being diagnosed with HIV wasn’t easy because I didn’t know whom to tell, because the nurse asked me to bring someone to be my monitor before I could start treatment. My family’s knowledge of HIV is very low based on some negative comments they normally make whenever they see persons with HIV on television. So, I couldn’t trust anyone for a long time until the nurse volunteered to be my monitor for me to start treatment”. Participant

### 2. Promoters of care engagement

Various factors encouraged or enabled GBMSM to partake in care and adhere to treatment. Such factors include the desire to live a healthy life, HIV counseling, detailed information on HIV medication, and positive experiences with healthcare providers.

#### Desire to live a healthy life.

The desire to be healthy and live a long life motivated the participants to start treatment and adhere. Perceiving both the risk of not taking ART as well as the benefits of ART adherence motivated GBMSM to engage in care:

“If I’m to stop the treatment, I think it is not going to help my health, so I had to continue with the treatment…It has helped me so well because I know friends who lost their lives for not going for treatment and me being in care and still here.” Participant

“For now, I will say this is who I am. After being diagnosed, I will say this is who I am, and I need to put myself on treatment. Make me live a little longer.” Participant

“Your life is in your hands; then you now determine how long you want to live by either taking it (ART) or not taking it. It is not easy because sometimes you feel like taking it, and sometimes you don’t feel like taking it. But, if that is the only way I can live among my peers, then I have no option but to take it.”

#### HIV counseling.

HIV-related counseling and information were crucial in supporting and reassuring GBMSM that they could have fruitful lives after HIV diagnosis. Providers encouraged participants to start treatment and to adhere to the treatment:

“They (provider) asked me to start treatment because for me knowing it (being positive), then it could be that it has been there for a long time, and I now know it, so the earlier I start treating it, the better so that I would still look healthy and okay.” Participant

“Since I know the consequence, and they have explained to me through counseling and stuff like that before even I begin medication. So, I try my possible best to stay fit and adhere to their rules so that I would also have a long life to live.” Participant

“The nurse called me after my first visit to advise me that she knows a lot of people who have been on HIV drugs for about 8 to 10 years, and they are healthy. Therefore, I should take my drugs and follow all the instructions. According to the Nurse, taking drugs will help me live my normal life and live long. This encouraged me to follow the steps.” Participant

#### Detailed medication information.

Clear and honest communication from healthcare providers about the treatment regimen and the potential possible side effects was critical to ensure adherence to ART. For some participants, the information and follow-up care aided in adherence with care or ART:

“We (participant and provider) normally talk about how I felt when I took the medications because they (providers) will give you a 3- or 4-month supply. So, they ask questions about your condition after your last visits to the facility. They also want to know how you reacted after taking the medications and if there were any complications.” Participant

“Well, he (the provider) told me that if I take my drugs on time and if I take it every day, my viral load will be low, so I think that one gave me hope so I never missed my drugs, and I am so grateful to God that I am undetectable till now. I started treating myself in 2017, and till now undetectable.” Participant

#### Positive experiences with healthcare providers.

For some GBMSM, supportive and affirmative care providers and the HCF environment encouraged them to adhere to care. They mentioned the friendliness and welcoming environment created by nurses who are friendly to GBMSM and people living with HIV (PLHIV). Participants described their experiences as judgment-free, which, in their opinion, encouraged them to stay in care:

“I used to think that once you contract the virus, you will be treated badly, but the experience was completely different. Their communication skills will make you feel comfortable and okay.” Participant

“I don’t miss my appointments. The hospital I go to for my medication takes good care of me, and they make sure I go for other tests and do some other checkups, which helps me a lot. With their service, it is so great. They encourage me a lot, the dos and don’ts about the HIV umbrella when you are positive, so I think it has helped me a lot.” Participant

### 3. Barriers to care engagement

Participants reported several barriers that impede their adherence to care. They include challenges taking ART, stigma and discrimination at the HCF and community, fear of disclosure and confidentiality at the HCF, substance use, health system challenges, use of alternative medication and spiritual healing, financial difficulty, and health insurance.

#### Challenges taking ART.

Initiating ART was overwhelming for many GBMSM. Some also found understanding the information providers gave about the medication challenging. In addition to adjusting to a once-daily pill regimen, taking the daily pill was a subtle reminder that they were living with HIV:

“At first, it was not a normal life because you know you don’t take medicine, and now you know you are going to take medicine. And taking medicine on a daily basis is not easy because it is not something you normally do… Sometimes, it’s difficult because it has to be a daily thing. It is not easy because sometimes you feel like taking it, and sometimes you don’t feel like taking it.” Participant

“The education given to me by the pharmacist was a whole lot. He told me I was going to experience a lot of complications after taking the ARV. I was contemplating not taking the drug.” Participant

#### Alternative medication and spiritual healing.

Instead of taking ART, some participants preferred to seek traditional medicine and/or spiritual healing in hopes of curing or eliminating their HIV. They mainly resorted to seeking advice or herbal remedies from spiritual churches, which discouraged the use of ART:

“He (friend) told me he went to see one pastor, and he advised him to stop taking ARV because it is going to shorten his life. He thinks that if he stops and lives a good lifestyle, he is going to be okay.” Participant

“I was trying to open up to someone. I needed help at that moment cos it got to a time when I was feeling very pale and very weak. So, I saw this advert on television that they had a cure for HIV and other diseases. It was a spiritual church. I went there; they did their herbal concoction and all that, but still, I don’t see any changes.” Participant

“I went to the church… they give you some drugs that will flush the virus. That’s what they will tell you. Oh, it will flush the virus, so I had absolute confidence within myself that I was going back to test negative again. It got to a time when they even told me to step on doves and chameleons. I stepped on the chameleon, and they said the chameleon would take the virus away from me.” Participant

#### Disclosure and confidentiality at HCF.

Participants were concerned about meeting family and friends at the HIV clinics within HCF. They avoided care because they feared unintentional disclosure that may arise from being seen by community members in the HIV section of HCFs (HIV services are typically in ART units in Ghana; hence, people mainly visit those departments if they are either living with HIV or going for a test).

“The government hospital (public HCF) was an open space, and I wasn’t that confident and more comfortable within myself because the people I live with were in there and people were passing. People could tell this is what was happening there, so with that place, No.” Participant

“It is expensive where I go (private HCF), but I still go because I feel comfortable with the place. I prefer it to where (public HCF) I will still go and get my medication with 5 cedis (about 70 cents) (cheaper place), and I will see so many familiar faces, and they will keep talking at my back (gossip)” Participant.

Some men were concerned about the lack of private rooms in facilities and the possibility of being overheard by others. Others were unsure providers would maintain confidentiality as some of their peers allegedly had experienced gossip at the HCF:

“Because of confidentiality, I can’t even have the zeal to share with you (provider) because I think that if I am sharing this with this person, another person will be listening to what I am saying. So, I will go back home with my problem. So, I think they should make enough room for confidentiality.” Participant

“A friend once told me he was at the [NAME] Hospital, and a doctor was gossiping about him. It led to a bad argument between them because he believed the doctor was gossiping about his HIV status or feminine characteristics to someone else and finger-pointing him. It was this same doctor who tested him when he was first diagnosed with HIV.” Participant

#### Family level stigma and discrimination.

Participants believed their communities and families were intolerant towards their sexual orientation and PLHIV. As such, they were afraid of being seen in the HCF and the possibility of family or friends finding out about their status from others:

“When you are feminine and gay in Africa and Ghana, it is different. It is something that you have to deal with personally. You have to have that strength to even live as a human being because your parents would be talking; you are too feminine; you are too soft, blah, blah, blah. So, socially, and mentally, you are having problems already. And getting information that you are positive and need treatment is another different issue, so you can imagine the two stresses you are going through.” Participant

“What happens if someone gets to see me here (HCF)? …that means it (HIV status) will start spreading, and the way information gets spread. Definitely, my parent will get to know about it. I am under their roof but now taking responsibility for myself. If they get to hear about this thing, they will take me out of their house without further explanation.” Participant

“You know everybody pointing fingers at you, you have HIV. It is so embarrassing. Some people are ignorant of HIV and AIDS, so they misunderstand HIV to be AIDS. So, they take it you have a deadly sickness, and they don’t want to come close to you. The sigma is high, to be honest with you. In my vicinity (community), it is really high. People pointing fingers at PLHIV, so you need also to be very careful with it (discrete).” Participant

#### Substance use.

Substance use and addiction impeded engagement with HIV care and adherence. Some participants had difficulty adjusting to taking ART as they used alcohol and other substances and were concerned about combining ART and other substances. For others, substance use sometimes prevented them from taking medication:

“As I said, my drug and alcohol addiction were the only things that may have prevented me from staying in care.” Participant

“I kept contemplating taking medication because I am the club type, and you know when you go to the club, you will Definitely smoke or drink. So, it was something that really scared me. I didn’t know if I should combine the medicine with the drugs and alcohol.” Participant

“At first, I used to drink a lot and smoke sometimes, and I was told by the nurse not to drink alcohol or smoke. I was addicted to drugs, so I was thinking about how I am going to combine the drugs and my medications.” Participant

#### Health system challenges.

There were challenges with long wait times and medication pick-up rules that restricted pick-up to specific days and units at the health facilities. These wait times or the ability to readily pick up medication affected engagement in care by increasing the time frame for accessing medication. In particular, some participants indicated that it was difficult to switch pick-up sites if they moved as they were constantly required to do routine health check-up tests and pick up medication from the same place. The lack of flexibility in medication pick-up location caused frustration and increased the time needed to pick up medication, yet moving is common as some move for work and others due to stigmatization in their communities:

“Well, treatment wasn’t convenient from the start because it was a government hospital (Public HCF), and it was cumbersome and time-consuming. You need to go three times and spend six hours every day at the facility. Go at dawn and join long queues to see a doctor. So, it was inconvenient, especially for me that was working early hours of the morning.” Participant

Due to my work, I travel a lot. There was a time I was in Tamale, and I used to take my medication over here (Accra). I had to fight for a transfer before getting my medication. It should be something you can get somebody you trust to pick it up for you. You can imagine what I was going through while in Tamale. I had to pick another form and go through another procedure over there. Start everything afresh. I tried to explain things, but they said I had to do a transfer. But I think they should plan it so that you can pick up your drugs anywhere you find yourself. People move out from vicinities because of stigma. Some go, and they don’t come back again.” (Participant)

“We are in the 21^st^ century. Things are being advanced in a way. Since HIV medication can be taken anywhere one finds himself, it doesn’t have to be one place where you have to constantly go and pick the drugs. I am at [facility NAME], but maybe I feel I don’t want to go to [other facility NAME]. I want to run my test and pick up my medication at [other facility NAME]. You have the card that indicates you are HIV; you should be able to take your drugs anywhere you find yourself. But this is not like that…sometimes, it affects people.” Participant

#### Financial barrier.

The cost of medication and transport to the health center to pick up the prescription made adherence challenging. Particularly for GBMSM who preferred to access HIV care away from their residential communities, traveling longer distances was costly. Thus, a lack of funds sometimes prevented the timely pick up of the medication:

“The only barrier sometimes is the finances. When you don’t have, or maybe it’s too far, you don’t have enough to go all the way, especially in my case where I don’t go to a center closer to me. So, imagine I don’t have that transport or money to buy my medicine; that is the only barrier.” Participant,

“Maybe my time is up, and I have to go for medication within that week or within that month, and I don’t have money. I wouldn’t be able to go there. But sometimes I tell them (provider) I have to come maybe this week, then I realize I wouldn’t be able to attend this week, then I will try them the following week. If they see I am not coming, they will get back like, ‘Why are you not still coming?’ then I could tell them that maybe it’s because of finances.” Participant,

“The only thing that might stop me from following up is money. It’s not always that you will have. If it (medication) is free, you can take it always, but at least now it is not free. With the three months (prescription), they would take 30 cedis (about 2.40 USD). So, imagine a week you don’t have, and you have to pick a car all the way from here to their place, then go all the way back home. If they are calculating, you should have around 40–45 cedis (5 USD) before you can still go there, so imagine that you don’t have, then obviously you won’t be able to attend it, then you would have to wait till you get that money.” Participant

Ghana’s National Health Insurance Scheme (NHIS) serves to guarantee access to HIV care, but its unavailability or lack of full coverage in private HCF, which participants expressed preference for, affects access to ART. Participants revealed that some providers changed their policies and did not accept public health insurance fully; hence, instead of a co-payment of 5 cedis, with the NHIS, some facilities require 30 cedis, thus exacerbating financial challenges.

“They (HCF) said now, they do not accept health insurance anymore. The health insurance used to cover it fully, so you would only pay 5 cedis, but now they don’t accept it. So, for all the three months you go, you have to pay for it.” Participant

“Now that they said they (HCF) are not taking insurance cards. That is the only thing. Because at first, when you know you have just 5 cedis, you can have your three months’ supply, but now it is 30 cedis. So, imagine you do not have 30 cedis, then it means you cannot go. That is the only thing that is not so comfortable.” Participants.

Crosschecking on the field from other persons living with HIV shows that these accounts are mainly true for private HCFs, as public facilities fully cover ART for free, but people avoid such places as indicated above due to fear of unintentional disclosure of HIV status and possible stigma.

## Discussion

This article provides qualitative accounts of experiences with HIV diagnoses and factors that promote or hinder access and ART adherence among GBMSM living with HIV in Ghana. Our findings provide important insights to inform interventions focusing on optimizing HIV outcomes among GBMSM in the country and elsewhere. We found that community and HCF-level HIV and sexual stigma, confidentiality issues, alternative medicine, and substance use remain the key barriers to care. Other barriers include healthcare system issues such as longer wait times and financial difficulties. Nonetheless, HCF -level factors such as positive experiences with healthcare providers, HIV counseling, and detailed medication information facilitate adherence to care among GBMSM.

Stigma remains the most salient factor that hinders adherence to care among GBMSM[9]–[12], [28]. Our findings show that stigma from HCF, as broadly reported elsewhere, and from family members and the community serve as barriers to the use of HIV services, including ART[9], [29]–[31]. Our participants reechoed sentiments from participants in the few available studies on HIV care among GBMSM in Ghana that point to the fear of being seen at the healthcare facility as a barrier to HIV care[12], [22], [32]. Due to family and community-level stigma, GBMSM need extreme confidentiality in HIV facilities and do not want people they know to meet or see them in facilities dedicated to HIV care. Similar to a previous report on HIV testing barriers in Ghana, GBMSM in this study feared that meeting a familiar person could spread rumors about their HIV status in the community, thus increasing stigma[10]. Therefore, strategies are needed to ensure HIV diagnoses, care visits, and medication pick-ups remain confidential. Some participants narrating their experience mentioned that they prefer private HCF because private they are more convenient and reduce the possibility of meeting familiar persons. This fear is possibly explained by the fact that public HCFs are free and, therefore, attract more people; like the rest of African countries, HIV services in Ghana are provided in HIV-specific departments; hence, people seen around the HIV departments remain associated with HIV-positive status[10], [23]. We therefore suggest using integrative care approaches that combine HIV services with other healthcare services instead of isolating them[33]. This method can make it harder for other facility users to identify those accessing HIV services and stop others from automatically identifying clients living with HIV. For example, by integrating HIV care into a chronic care clinic that also provides services for hypertension and diabetes, we can enhance the privacy and confidentiality of HIV services and decrease the negative stereotypes associated with HIV. Indeed, this process has been shown to reduce such stigma and fear of breach of confidentiality in another African country[33]

This study further shows that mistreatment and stigmatizing experiences of GBMSM directly from healthcare providers can also affect adherence among GBMSM in Ghana. As indicated by participants, some providers do not respect them due to their backgrounds and some gossip about them to their colleagues, thus affecting their interest in visiting the facilities. The findings are consistent with other studies that show that negative healthcare provider attitudes and stigma associated with sexual behavior and HIV status affect testing, linkage, and adherence to care among GBMSM[4], [9]–[12], [14]. Conversely, our analysis of promoters of HIV care adherence among GBMSM suggests that providers who are friendly and judgment-free help GBMSM feel comfortable going for care. This finding conforms with previous studies that point to friendly providers as key to maintaining HIV care adherence[10], [34], [35]. Also, recent studies in Ghana show that GBMSM prefer HIV testing from friendly providers and other GBMSM-led organizations to avoid stigma[10], [31]. Thus, interventions are urgently needed that address healthcare-level sexual and HIV stigma and promote a safe and friendly HIV care environment for GBMSM.

Improving the care environment is essential, especially in Ghana, where alternative medicine through religious bodies and herbal medicine providers remains common and advertised as a legitimate cure for HIV[36]–[38]. As reported, some participants saw TV advertisements and went to churches where they obtained potions supposed to treat HIV. The use of such untested medications, even if not hazardous for the person, will not help in treating HIV[39]. It also explains why many do not receive treatment until the point of severe illness and the high mortality rates among persons living with HIV in West Africa and SSA [40]–[43]. These findings support previous findings in SSA and lay bare the underlying need to address HIV stigma and increase HIV education among high-risk groups, PLHIV, and the general community [10], [23], [44], [45]. Specifically, interventions need to address HIV misinformation and disinformation to combat the association of HIV and death, as that leads to distress and lack of acceptance of HIV status, as well as seeking alternative ways such as religious, spiritual, and herbal medicine in the case of these participants.

This study also provides early findings to show a possible link between substance use and low adherence to HIV medication among GBMSM living with HIV in Ghana. Some participants mentioned that they used alcohol and drugs and partied a lot before being diagnosed with HIV, indicating that they delayed or contemplated delaying medication due to their drug use. This finding, though early, corresponds with a few studies from Ghana and elsewhere showing that substance use can negatively impact medication adherence and increased risk behaviors among PLHIV[46], [47]. It also highlights the importance of further studies in Ghana to provide additional insights into this phenomenon and possible interventions to address HIV and substance use.

Beyond individual and HCF level factors, health system issues and structural level factors such as health insurance and lack of funds also affect access to care for GBMSM living with HIV in Ghana. As shown by participant accounts, PLWHIV have to go to the same HIV clinic for medication and cannot move from one clinic to another, which places challenges when traveling and increases longer wait times for medication pick-up. Although not inexpensive, the charges associated with taking medication and transportation increased costs, especially in private facilities (usually located in distant places), which participants prefer due to the privacy[10], [33]. Therefore, policymakers need to consider alternative ways to provide access to reduce travel and time spent getting medications. Decentralizing pickup points to include places such as local community pharmacies and allowing persons to choose their pharmacy will help reduce transportation challenges and time spent accessing medication.

While this study provides essential insights into HIV care engagement among GBMSM living with HIV, the findings should be considered in light of broader GBMSM research in Ghana and the region. The sample included only a small number of GBMSM living with HIV. Community organizations that link GBMSM to HIV care in Accra and Kumasi recruited the participants for this study. Thus, the findings may not reflect the experiences of other GBMSM living with HIV who belong to different networks, not in networks, or in different communities across Ghana and West Africa. Investigations must examine the promoters and barriers to care, especially exploring the early findings, such as substance use and its impact on care, family-level stigma, and use of alternative medicine among GBMSM living with HIV in Ghana and West Africa.

## Data Availability

The datasets generated and analyzed during the current study are not publicly available due to the high risk of persecution and severe adverse social consequences related to the socio-political sensitivity of the topic of same-sex behaviors in Ghana; however, data are available from the corresponding author on reasonable request.
